# Friend or Foe – Tc17 cell generation and current evidence for their importance in human disease

**DOI:** 10.1093/discim/kyad010

**Published:** 2023-07-20

**Authors:** Anna Veronika Hipp, Bertram Bengsch, Anna-Maria Globig

**Affiliations:** Clinic for Internal Medicine II, Gastroenterology, Hepatology, Endocrinology, and Infectious Diseases, University Medical Center Freiburg, Faculty of Medicine, Freiburg, Germany; Clinic for Internal Medicine II, Gastroenterology, Hepatology, Endocrinology, and Infectious Diseases, University Medical Center Freiburg, Faculty of Medicine, Freiburg, Germany; Clinic for Internal Medicine II, Gastroenterology, Hepatology, Endocrinology, and Infectious Diseases, University Medical Center Freiburg, Faculty of Medicine, Freiburg, Germany

**Keywords:** T cell, IL-17, Tc17, Th17, inflammatory bowel disease

## Abstract

The term *Tc17 cells* refers to interleukin 17 (IL-17)-producing CD8^+^ T cells. While IL-17 is an important mediator of mucosal defense, it is also centrally involved in driving the inflammatory response in immune-mediated diseases, such as psoriasis, multiple sclerosis, and inflammatory bowel disease. In this review, we aim to gather the current knowledge on the phenotypic and transcriptional profile, the *in vitro* and *in vivo* generation of Tc17 cells, and the evidence pointing towards a relevant role of Tc17 cells in human diseases such as infectious diseases, cancer, and immune-mediated diseases.

## Introduction

T cells are a central component of the adaptive immune system. They can recognize and respond to specific antigens, eliminate infected or cancerous cells, and generate memory responses that protect the host from reinfection. T cells can be divided into several subtypes, including CD4^+^ T cells and CD8^+^ T cells. CD4^+^ T cells, also called T helper cells, coordinate the immune response by releasing cytokines that activate and recruit other immune cells. Specific cytokines can shape different immune responses: Type 1 responses are mediated by interferon-γ (IFN-γ) and tumor necrosis factor-α (TNF-α)-producing *Th1* cells and protect against intracellular pathogens, but are also associated with immune-mediated diseases. Type 2 responses involve interleukin (IL)-4, IL-5, and IL-13-producing *Th2* cells and the activation of eosinophils, basophils, and mast cells as well as the production of immunoglobulin E, thus protecting against helminth infections. On the flip side, overreaching type 2 immunity is observed in allergic diseases. The more recently described type 17 immunity comprising the *Th17* cell-secreted cytokines IL-17 and IL-22 recruits neutrophils and mononuclear phagocytes and induces antimicrobial responses by epithelial cells, thereby providing defense against fungal infections. Overreaching type 17 responses have, however, been implicated in the pathogenesis of immune-mediated diseases such as multiple sclerosis (MS) and inflammatory bowel disease (IBD) [1].

A major role for CD8^+^ T cells is to recognize presented antigenic peptides via their T cell receptor (TCR) and subsequently kill virus-infected or cancerous cells. This is a function of cytotoxic *Tc1* cells. However, CD8^+^ T cells can also produce different cytokines and perform functions other than killing. In the last decades, similar to CD4^+^ T cell nomenclature, additional distinct CD8^+^ T cell subtypes like *Tc2*, *Tc9*, CD8^+^ T regulatory cells, and *Tc17* cells have been identified and characterized [2, 3]. Among these specialized CD8^+^ T cell subsets, Tc17 cells have mainly been identified based on their ability to produce type 17 cytokines such as IL-17A and IL-17F. Both in healthy and diseased humans, most Tc17 cells have been found to produce IL-17A, but only about 20% of Tc17 cells coproduce IL-17F [4]. Besides the production of their namesake cytokine IL-17, Tc17 cells are also capable of producing additional cytokines, such as IFN-γ, TNF-α, GM-CSF, IL-21, and IL-22 [5]. Quantitatively, about 90% of Tc17 cells can coproduce TNF-α and about half of Tc17 cells can coproduce IFN-γ in healthy donors. In inflammatory conditions like active IBD, this coproduction profile remains unchanged, suggesting it is an inherent feature of the Tc17 program [4].

The IL-17 receptor is universally expressed by epithelial cells and stromal cells such as fibroblasts [6–8], which upon IL-17 stimulation, produce cytokines and chemokines such as IL-6, CXCL2, and CXCL8 and the hematopoietic factors G-CSF and GM-CSF, thereby enhancing the local and systemic immune response, e.g. by recruitment of neutrophils [9–12]. Tc17 cells have been implicated in a variety of diseases such as viral, bacterial, and fungal infections, gastrointestinal malignancies, and immune-mediated diseases of the skin, the gastrointestinal tract, and the central nervous system, and the IL-17 pathway has recently become a prominent target of novel biological therapies [6, 13, 14]. In this review, we will summarize the current knowledge on the phenotypic and transcriptional profile of Tc17 cells, their *in vitro* and *in vivo* generation, and their involvement in human diseases.

### The IL-17 cytokine family

The IL-17 cytokine family includes six members: IL-17 (also called IL-17A), IL-17B, IL-17C, IL-17D, IL-17E (also called IL-25), and IL-17F [15]. IL-17A and IL-17F share the greatest homology and are the best-characterized family members [16–18]. Both induce the production of proinflammatory cytokines and chemokines in target cells, thereby leading to inflammation and recruitment of neutrophils. Less is known about IL-17B and IL-17C, although both are thought to also mediate the inflammatory response and be involved in arthritis [19]. IL-17E is the most unique family member and can regulate type 2 immune responses [15].

### Tc17 cell phenotype

**Figure 1:**
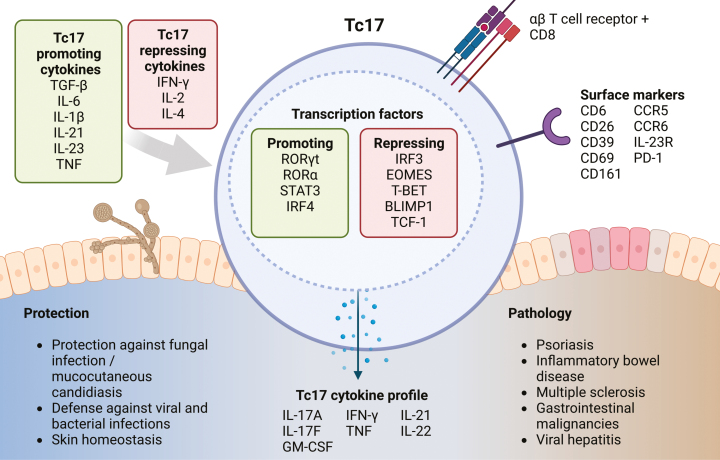
Key features of Tc17 cells in protection and pathology

Tc17 cells share key transcriptional, phenotypic, and functional features with their Th17 counterparts, including the expression of the type 17 master regulator retinoic acid receptor-related orphan receptor γt (RORγt) [20–23]. They are further characterized by the expression of a variety of surface markers, such as the C-type lectin-like receptor CD161, the dipeptidyl peptidase IV (CD26), and the CC motif chemokine receptors CCR5 and CCR6 [24–28] (**Figure 1**). Tc17 cells have been found to be mostly part of the CD27^-/+^ CD28^+^ CD45RA^-^ cell subsets in humans [28, 29]. These markers can help identify Tc17 cells in diagnostic and biomarker settings that do not allow for intracellular analysis.

CD161 is expressed on human circulating lymphocytes able to produce IL-17, and its expression can be induced by expression of the type 17 master transcription factor *RORC* in naïve T cells [30], supporting the close correlation between CD161 expression and type 17 programming. While the binding of CD161 to its ligand lectin-like transcript 1 (LLT1) has costimulatory effects on natural killer (NK) cells, the role of CD161:LLT1 in modulating the function of T cells remains unclear, in particular, it remains unclear if LLT1 modulates IL-17 production [31–33].

CD26 was initially described as a T cell activation marker and regulates T cell proliferation [34, 35]. It acts as an ectoenzyme that cleaves amino-terminal dipeptides from different chemokines [36]. CD26 expressed on IL-17-producing human T cells is enzymatically active and negatively regulates the chemotactic T cell response, which can be restored by inhibition of CD26 [24].

The expression of CCR5 and CCR6 is shared among both Tc17 and Th17 cells [28, 37, 38]. Mechanistically, the interaction of CCR6 and its ligand CCL20 plays an important role for type 17 T cell migration to epithelial sites such as the intestinal epithelium [39, 40] or to the infected lung [41], as well as for T cell localization close to the intrahepatic bile ducts in inflammatory liver diseases [42].

While the expression of none of these markers is exclusive to Tc17 cells per se, a signature of seven surface markers uniquely expressed by human Tc17 cells has recently been identified by our group and consists of a high expression of CD161 and CD26, in addition to CD6, CD69, CD39, PD-1, and a low expression of CD27 [4].

CD6 is involved in the fine-tuning of T cell activation signals [43] and T cell proliferation [44]. Its ligands include activated leukocyte cell adhesion molecule (ALCAM/CD166) and CD318 [45–47]. The cytosolic part of CD6 forms a complex signalosome and can both positively and negatively regulate T cell activation [43, 48, 49]. Importantly, inhibition of CD6 on human CD8^+^ T cells results in reduced production of IL-17A, IFN-γ, and TNF-α [4]. It has previously been suggested that anti-CD6 antibodies modulate activation and differentiation of Th17 cells through modulation of signal transducer and activator of transcription 3 (STAT3) signaling [50], however, we were not able to reproduce this in Tc17 cells [4].

An increase in the expression of the type II transmembrane protein of the C-type lectin family CD69 can be rapidly detected after stimulation of different immune cell types including T cells [51] and CD69 is consequently commonly used as a marker for cell activation. Deletion of CD69 leads to alterations in the disease activity in arthritis and colitis mouse models [52–55], supporting a functional role of CD69-signaling in the regulation of Th17 differentiation and production of cytokines [56, 57], although the reports regarding the question whether CD69 deletion is clinically beneficial in these disease contexts are contradictory. Interaction of CD69 with its ligand galectin-1 impairs type 17 differentiation of both human and murine naïve CD4^+^ T cells, an effect which is reversed by anti-CD69 antibodies [58]. Mechanistically, the cytoplasmic fraction of CD69 interacts with the JAK3/STAT5 pathway, resulting in the suppression of STAT3-mediated type 17 gene expression [57, 59]. In addition, CD69 can also associate with the sphingosine-1-phosphate receptor 1 (S1PR1) and the heterodimeric amino acid transporter complex LAT1/CD98 [60, 61]. The association with CD69 prevents S1PR1 from mediating tissue egress of T cells [60, 62]. Consequently, CD69 is a common marker for tissue-resident memory cells, which share some characteristics with Tc17 cells and can produce IL-17 [63].

The ectoenzyme CD39 (ENTPD1/ectonucleoside triphosphate diphosphorylase 1) catalyzes the extracellular conversion of adenosine triphosphate (ATP) and adenosine diphosphate to adenosine monophosphate [64, 65], which is then converted to adenosine by CD73 [64, 66, 67]. The activity of these two enzymes thereby regulates the balance between extracellular proinflammatory ATP and its immunosuppressive derivates. CD39 expressed on CD8^+^ T cells is enzymatically active and negatively regulates their cytokine production [68]. While the frequency of CD39^+^ cells in the peripheral blood is typically low in both healthy donors and cancer patients, an increased expression has been described for tumor-infiltrating CD8^+^ T cells [69, 70] and hepatitis C virus (HCV)- and HIV-specific CD8^+^ T cells [71]. CD39^+^ CD8^+^ T cells recognize tumor antigens [72] and display features of T cell exhaustion such as an increased expression of inhibitory receptors and impaired production of TNF-α and IL-2 [70]. CD39 is further involved in the pathogenesis of IBD, as polymorphisms in the *ENTPD1* gene are associated with increased susceptibility to Crohn’s disease in humans and CD39-deficiency exacerbates disease severity in murine IBD models [68, 73].

Programmed cell death protein 1 (PD-1) is an inhibitory receptor expressed by various immune cells including T cells, whose ligands include PD-L1 and PD-L2. PD-1 engagement results in the reduction of downstream TCR and co-stimulatory signaling [74–76] and curtails the production of cytokines and T cell proliferation [77–80]. An increased expression of PD-1 can be detected on activated T cells and is, besides its induction upon T cell activation, also linked to T cell differentiation status [81–83]. Activity of PD-1 is vital for the prevention of autoimmunity and the maintenance of self-tolerance [84, 85]. In cancer and chronic viral infection, PD-1 signaling is critical for the development of T cell exhaustion [86–90]. Conversely, the blockade of PD-1 and other inhibitory receptors via therapeutic antibodies (a strategy termed *immune checkpoint blockade*) has been a breakthrough in cancer therapy [91–93].

In the chronic inflammatory skin disease psoriasis, the PD-1^+^ subset of the CD103^+^ CD8^+^ skin-infiltrating cells was shown to preferentially produce IL-17A compared to its PD-1^-^ counterpart and the frequency of these PD-1^+^ CD103^+^ CD8^+^ cells could be correlated with disease severity [94]. Another recent study confirmed a high expression of inhibitory receptors by skin-infiltrating Tc17 cells in psoriasis and while these cells exhibited some features of dysfunction, their transcriptional profile was distinct from melanoma-infiltrating, *bona fide* exhausted CD8^+^ T cells [95].

CD27 is a transmembrane homodimer and part of the TNF-receptor family with costimulatory effects on T cell responses [96–98]. CD27-mediated costimulation is relevant for the naïve, effector, and memory stages of T cell differentiation [98–102]. Its ligand CD70 is, within immune cells, expressed on APCs, DCs, B cells, and T cells [98]. CD27 exerts its costimulatory effects through the activation of canonical and alternative nuclear factor κB (NFκB) pathways [97, 103] and has been shown to increase the expression of the anti-apoptotic molecule BCL-x_L_ on CD4^+^ T cells [104]. Clinically, CD27 stimulation has been shown to synergize with PD-1 blockade to increase CD8^+^ T cell expansion and effector function in cancer immunotherapy [105–107].

Despite Th17 cells sharing the expression of CD161, CD26, CD6, and CD69 with their CD8^+^ T cell counterparts, CD27, PD-1, and CD39 were not found to be similarly differentially expressed in Th17 cells, suggesting that albeit Th17 and Tc17 cells share common characteristics, they also exhibit cell-type specific phenotypic features [3, 4, 24].

### Diversity of IL-17-producing CD8^+^ cells

In addition to Tc17 cells that express a conventional TCR consisting of an α- and a β-chain and that recognize peptide-major histocompatibility complex (MHC)-I complexes, other types of IL-17A^+^ CD8^+^ cells comprise mucosa-associated invariant T cells (*MAIT cells*), *γδ T cells*, and natural killer T cells (*NKT cells*) [4]. Innate-like MAIT cells are MHC-related protein 1-restricted and express a semi-invariant αβ-TCR composed of a Vα7.2 and a TCR-β chain. Interestingly, MAIT cells in their final stage of development within the thymus can be subdivided into MAIT1 and MAIT17 cells, the latter of which share a transcriptional program closely related to the type 17 program of Tc17 and Th17 cells [108].

Unconventional γδ T cells express a namesake TCR composed of one TCR γ-chain and one TCR δ-chain and combine features of innate and adaptive immunity that allow them to act as the first line of defense during early phases of inflammation. A subset of γδ T cells, *γδ 17 T cells*, is able to rapidly produce IL-17A after pathogen contact, preferentially at barrier sites such as the skin or mucosal tissues [109, 110].

In the peripheral blood of healthy donors, the frequency of IL-17A^+^ CD8^+^ T cells is below 2% of CD8^+^ T cells, with significant increases in inflammatory diseases [4, 28, 111]. Comparing the different sources of IL-17A production, both MAIT cells and Tc17 cells make up the majority of IL-17A^+^ CD8^+^ T cells in healthy humans, followed by NKT cells and γδ T cells [4]. In an inflammatory setting, such as Crohn’s disease, the ratio of MAIT cells to conventional Tc17 cells shifts towards significantly more conventional Tc17 cells, suggesting that conventional Tc17 cells in particular drive the inflammatory pathogenesis [4]. Importantly, despite the fact that these IL-17-producing cell types share many common features, their differentiation also requires very distinct transcriptional adaptations, such as different dependence on interferon-regulatory factor 4 (IRF4), T cell factor-1 (TCF-1), and promyelocytic leukemia zinc finger [3, 112]. Prior work has largely relied on identifying IL-17-producing cells purely based on their production of IL-17 but has not necessarily distinguished these cells into the aforementioned conventional and unconventional CD8^+^ cell subsets. This might explain why the understanding of the protective vs. pathogenic role of Tc17 cells is still incomplete. More in-depth phenotyping of the IL-17-producing cell subsets and distinction between them in a variety of health and disease settings will help elucidate the pathogenic roles of the individual cell types.

## Generation of Tc17 cells and key transcription factors

### *In vitro* generation of Tc17 cells

CD8^+^ T cell differentiation is tightly regulated via a complex network of cytokines and lineage-defining transcription factors [113, 114]. While the exact requirements for Tc17 cell differentiation are still unknown, several lessons have been learned from human and murine *in vitro* and *in vivo* systems.

Based on evidence that specific cytokines such as TGF-β, IL-1β, IL-6, IL-21, and IL-23 are important for the generation of CD4^+^ Th17 cells [115–126], similar cytokines have been used to establish *in vitro* type 17 polarizing conditions (**Table 1**). These *in vitro* differentiation protocols have been used for the generation of both human and murine Tc17 cells. However, the overall *in vitro* human Tc17 cell yield in the literature has been low compared to the Tc17 frequencies achieved with *in vitro* differentiation protocols using murine cells, much like for Th17 cells [4, 28, 127–139]. The frequencies of IL-17-producing CD8^+^ T cells achieved under *in vitro* conditions using human cells range from 0.1% to 6% of CD8^+^ T cells, whereas up to 80% could be reached using murine T cells (**Table 1**). The frequencies of IL-17-producing cells achieved using different *in vitro* culture protocols are variable, but nevertheless some important principles and differences between *in vitro* and *in vivo* generation of Tc17 cells can be concluded.

**Table 1:** Published *in vitro* culture conditions for generation of Tc17 cells

Cell type	*In vitro* culture conditions^*^	Tc17 yield	Publication
**Human**			
naïve CD8^+^ T cells	Culture for 5 days with anti-CD3 (2 µg/ml)/CD28 (1 µg/ml) + IL-1β IL-6 IL-23 TGF-β, further 4 days of culture with IL-2 (200 U/ml)	0.11% IL-17A^+^ of CD8^+^ T cells	Kondo/Takata, *J Immunol* 2009 [28]
CD8^+^ T cells	Culture for 3 days with anti-CD3 (2 µg/ml)/CD28 (0.5 µg/ml) + IL-6 (50 ng/ml) TGF-β1 (10 ng/ml)	25 pg/ml IL-17A (detected by ELISA)	Gras, *Molecular Pharmaceutics* 2012 [127]
Naïve CD8^+^ T cells	Culture for 7 days with anti-CD3 (0.5 µg/ml)/CD28 (0.5 µg/ml) + IL-1β (10 ng/ml) IL-6 (50 ng/ml) TGF-β1 (10 ng/ml) anti-IL-4 (7 µg/ml) anti-IFN-γ (7 µg/ml)	1.9% IL-17^+^ of CD8^+^ T cells	Renavikar, *Front Immunol* 2020 [128]
PBMCs	Culture for 3 days with anti-CD3/CD28 + IL-1β (12.5 ng/ml) IL-6 (25 ng/ml) IL-23 (25 ng/ml) TGF-β (5 ng/ml)	6.06% IL-17A^+^ of CD8^+^ T cells	Globig/Hipp, *Nat Commun* 2022 [4]
**Mouse**			
Hapten-primed CD8^+^ T cells	Culture for 2 days with hapten-labeled bone-marrow-derived dendritic cells + IL-23 (5 ng/ml)	1.4 ng/ml IL-17A (detected by ELISA), 1.5% IL-17A^+^ of CD8^+^ T cells	He, *J Immunol* 2006 [129]
Mixed splenocytes from BALB/c and C57BL/6 mice	Culture for 5 days + IL-6 (100 ng/ml) TGF-β (1 ng/ml)	32.8% IL-17A^+^ of CD8^+^ T cells	Liu/Tsai, *J Leukoc Biol* 2007 [130]
WT OT-1 and IFN-γ^-/-^ OT-1 CD8^+^ T cells	Culture for 4 days with peptide-pulsed B cell blasts + IL-2 (4.7 ng/ml) IL-1β (10 ng/ml) IL-6 (20 ng/ml) TGF-β (3 ng/ml) anti-IL-4 (10 µg/ml) anti-IFN-γ (10 µg/ml) IL-21 (80 ng/ml) IL-23 (50 ng/ml)	WT OT-1: 45% IL-17A^+^ of CD8^+^ T cells IFN-γ^-/-^ OT-1: 60% IL-17A^+^ of CD8^+^ T cells	Hamada, *J Immunol* 2009 [131]
Naïve CD8^+^ T cells	Culture for 2 days with peptide-pulsed-irradiated splenocytes + IL-1β (20 ng/ml) IL-6 (20 ng/ml) TGF-β (5 ng/ml) IL-23 (20 ng/ml) anti-IFN-γ (10 µg/ml) anti-IL-4 (10 µg/ml), further 3 days of resting	43% IL-17A^+^ of CD8^+^ T cells	Yen, *J Immunol* 2009 [132]
CD8^+^ T cells	Culture for 3 days with anti-CD3 (1 µg/ml)/CD28 (1 µg/ml) + TGF-β1 (2 ng/ml) IL-6 (20 ng/ml) anti-IFN-γ (5µg/ml) anti-IL-2	70% IL-17A^+^ of CD8^+^ T cells	Ciric, *J Immunol* 2009 [133]
Naïve P14 CD8^+^ T cells	Culture for 5 days with GP_33-41_ peptide (300 ng/ml) + IL-6 (20 ng/ml) TGF-β1 (5 ng/ml) IL-23 (10 ng/ml) anti-IFN-γ anti-IL-4	56% IL-17A^+^ of CD8^+^ T cells	Curtis, *J Immunol* 2009 [134]
CD8^+^ T cells	Culture for 3 days with anti-CD3 (5 µg/ml)/CD28 (1.5 µg/ml) + TGF-β (2 ng/ml) IL-6 (50 ng/ml) or IL-21 (50 ng/ml)	30% IL-17A^+^ of CD8^+^ T cells	Huber, *Eur J Immunol* 2009 [135]
CD8^+^ T cells	Culture for 3 days with anti-CD3 (4 µg/ml)/CD28 (1 µg/ml) and CD8^+^-depleted-irradiated splenocytes + TGF-β1 (2 ng/ml) IL-6 (100 ng/ml) anti-IFN-γ (10 µg/ml), further culture for 2 days with IL-2 (20 U/ml)	35% IL-17A^+^ of CD8^+^ T cells	Yeh/Glosson, *J Immunol* 2010 [136]
CD8^+^ T cells	Culture for 3 days with anti-CD3 (3 µg/ml)/CD28 (0.5 µg/ml) + IL-6 (20 ng/ml) TGF-β1 (0.5 ng/ml) IL-2 (50 U/ml) anti-IFN-γ (5 µg/ml)	18.8% IL-17A^+^ of CD8^+^ T cells	Huber, *J Clin Invest* 2013 [137]
CD8^+^ T cells	Culture for 4 days with anti-CD3 (1 µg/ml) and antigen-presenting cells + IL-6 (20 ng/ml) TGF-β (2 ng/ml) anti-IFN-γ (5 µg/ml)	9 ng/ml IL-17A (detected by ELISA) 82% IL-17A^+^ of CD8^+^ T cells	Flores-Santibáñez, *Immunology* 2015 [138]
CD8^+^ T cells	Culture for 3 days with SIINFEKL (OVA_257–264_) peptide (0.5 µg/ml) and CD90-depleted WT splenocytes (for OT-1) or anti-CD3 (3 µg/ml)/CD28 (0.25-4 µg/ml) (for WT B6) + IL-6 (10 ng/ml) TGF-β (2 ng/ml) IL-23 (25 ng/ml) IL-1β (5 ng/ml) anti-IFN-γ (10 µg/ml)	55% IL-17A^+^ of CD8^+^ T cells	Arra, *Oncoimmunology* 2017 [139]

^*^The condition with the highest Tc17 yield was listed when multiple type 17 polarizing conditions were used.

TGF-β is a common component of cytokine cocktails for the *in vitro* generation of Tc17 cells, but it has been shown to be dispensable for Tc17 cell generation *in vivo*: Transgenic animals carrying a dominant negative form of the TGF-β receptor II are still able to generate IL-17^+^ CD8^+^ T cells (but fail to generate IL-17^+^ CD4^+^ T cells), and neutralization of TGF-β only has little effect on Tc17 frequencies, highlighting different requirements for CD8^+^ and CD4^+^ IL-17^+^ T cell differentiation [140, 141]. The cellular requirement for IL-6 signaling to induce a Tc17 program depends on the cell’s activation status: While IL-6 is frequently used in *in vitro* differentiation cocktails for the generation of Tc17 cells from naïve CD8^+^ T cells, it might not be relevant for the conversion of already differentiated murine Tc1 cells into Tc17 cells [142]. This is likely due to the expression pattern of the IL-6 receptor, which is expressed on naïve, but not on activated CD8^+^ T cells [142]. Intracellular effects of IL-6, analyzed in murine CD4^+^ T cell differentiation, include a RORγt-independent upregulation of the *Il21* gene, and RORγt-dependent effects such as the expression of *Il17* [124]. *In vitro* addition of IL-21 increases IL-17A production by murine CD8^+^ T cells and further supports Tc17 polarization in combination protocols with other cytokines [131, 133, 135]. In turn, murine and human Tc17 cells themselves also produce IL-21 [143] and for Th17 cells, autocrine stimulation by IL-21 has been suggested [122, 124, 141]. Addition of IL-23 alone during activation with peptide-presenting APCs has been shown to be sufficient for *in vitro* induction of IL-17A production and increased expression of RORγt and RORα by some murine CD8^+^ T cells. This could be further enhanced by the addition of IL-6 and TGF-β [134]. Furthermore, IL-23 has been shown to be the critical signal for IL-17 production in response to *Klebsiella pneumoniae* infection in mice [144]. While IL-1β is a common component of the cytokine cocktails used for *in vitro* generation of Th17 cells, its addition only led to a minor increase in murine IL-17A-producing CD8^+^ T cells in one study and showed no effects in a second study [131, 132]. Despite these modest effects, it has been included in the cytokine cocktails used for *in vitro* Tc17 cell generation by several groups (**Table 1**).

Results from *in vitro* CD4^+^ T cell differentiation experiments suggest a sequential role of the aforementioned cytokines, as IL-6 appears to initially program Th17 cell differentiation together with TGF-β; and IL-21 and IL-23 act in subsequent sequential pathways that amplify Th17 programming [17, 124]. However, the applicability of this concept to CD8^+^ T cells is currently still unclear, particularly since the expression of the IL-23R subunits differs on naïve CD8^+^ T cells and naïve CD4^+^ T cells [145] and the effects of *in vitro* addition of IL-23 alone during activation of naïve murine CD8^+^ T cells are inconsistent [133, 134, 145]. IL-23 has also been shown to promote IL-22 production by murine Tc17 cells and alters the pathogenic capacity of Tc17 cells [133]. The addition of both TGF-β and IL-6 during activation of naïve murine CD8^+^ T cells induces IL-17 production with a small proportion of IFN-γ co-producing cells [130, 133]. In contrast to these Tc17-promoting cytokines, IFN-γ and IL-2 have been shown to suppress murine and human Tc17 differentiation [128, 131–133, 146, 147].

### Transcriptional landscape of Tc17 cells

Most evidence for the transcriptional prerequisites of Tc17 programming stems from mouse models and **Table 2** compares the key phenotypic, transcriptional, and functional features of murine and human Tc17 cells, based on the publications analyzed for this review.

**Table 2:** Published features of human and murine Tc17 cells

	Human	Mouse
**Tc17-promoting cytokines**	TGF-β, IL-6, IL-1β, IL-23	TGF-β, IL-6, IL-1β, IL-23, IL-21, TNF-α
**Tc17-repressing cytokines**	IFN-γ, IL-4	IFN-γ, IL-4, IL-2
**Tc17-promoting transcription factors**	RORγt, STAT3	RORγt, STAT3, IRF4
**Tc 17-repressing transcription factors**		IRF3, EOMES, T-BET, BLIMP1, TCF-1
**Tc17 effector functions**	IL-17A, IL-17F, IFN-γ, TNF-α, IL-22, XCL-1	IL-17A, IL-17F, IFN-γ, TNF-α, GM-CSF, IL-13, IL-21, IL-22, IL-5
**Tc17 surface markers**	CCR5, CCR6, IL-23R CD26, CD161, CD6, CD69, CD39, PD-1	CCR6, IL-23R

Both IL-6 and IL-21 are potent activators of STAT3. Indeed, similar to Th17 cells that rely on the activity of STAT3 [21, 22, 124], STAT3 activity has also been found to be essential for the generation of murine Tc17 cells [132, 135, 143] and is upregulated in human Tc17 cells (**Table 2**) [4]. Mechanistically, activation of STAT3 is crucial for the expression of the type 17 master regulators of the ROR family, RORα and RORγt, and STAT3 has been found to direct the molecular switch between type 17 and cytotoxic lymphocyte differentiation programs in murine cells [20–23, 135]. Furthermore, cytotoxic T-lymphocyte-associated protein 4 (CTLA-4) has been identified as a stabilizing factor of IL-17 production and Tc17 differentiation via control of STAT3 activity. Consequently, murine CTLA-4-deficient T cells express low levels of type 17 signature molecules [139]. In contrast to the Tc17-promoting role of STAT3, STAT5 has been found to repress IL-17 production in both murine CD4^+^ Th17 and CD8^+^ Tc17 cells [146].

The orphan nuclear receptor RORγt is required for the differentiation of murine Th17 cells and the expression of the *Il17a* and *Il17f* genes [23]. While RORγt is also highly expressed in both murine and human Tc17 cells and, by binding to the IL-17 conserved noncoding sequence-2-enhancer, increases IL-17 production in CD8^+^ T cells, its overexpression alone in murine CD8^+^ T cells under non-Tc17 polarizing conditions only results in a minor increase in Tc17 cells [3, 135, 148], indicating additional transcriptional changes are needed for induction of Tc17 cells.

The downregulation of T-box transcription factors T-BET and EOMES, or T-BET and BLIMP-1 is closely correlated with the capacity of murine CD8^+^ T cells to produce IL-17 [134, 135, 147, 149–151]. Interestingly, *Tbx21* and *Eomes* murine double knockout (KO) cells, but not *Tbx21* or *Eomes* single KO CD8^+^ T cells, upregulate mRNA levels of the type 17 markers *Rorc*, *Il23r*, *Il17a*, *Il21*, and *Il22* [151].

The transcription factor aryl hydrocarbon receptor (AhR) is upregulated in murine and human Th17 cells [152]. Inhibition or deletion of AhR during *in vitro* polarization impairs the production of IL-17A and abrogates the production of IL-22 by murine Th17 cells [152, 153], effects which imply that AhR is especially important for more plastic Th17 cell populations [154]. Mechanistic studies using murine CD4^+^ T cells under Th17 polarizing conditions show that AhR inhibits STAT1 [155] and reduces IL-2 expression by induction of *Ikzf3*, encoding the transcription factor AIOLOS [156]. During *in vitro* culture of murine Th17 cells, sustained AhR expression was dependent on the presence of TGF-β1 [157]. Genetic deletion or inhibition of AhR also impaired murine Tc17 cell development, but, unlike in CD4^+^ Th17 cells, binding of AhR to its ligand 6-formylindolo[3,2-b]carbazole (FICZ) did not increase IL-17A production in AhR^+/-^ mouse CD8^+^ Tc17 cells [153]. These data suggest that, unlike in Th17 cells, the baseline activity of AhR might suffice for the induction of murine Tc17 cells.

Other orchestrators of Tc17 cell differentiation include *Tcf7*, *Irf3*, and *Irf4*: A loss of *Tcf7* in murine cells modulates chromatin accessibility with increased accessibility of the *Rorc*, *Il17a*, and *Il17f* genes, and reduced accessibility of the *Tbx21*, *Eomes*, and *Irf4* loci [3]. Furthermore, *Tcf7* suppresses Tc17 differentiation via suppression of the MAF (MAF BZIP transcription factor) – RORγt axis [3]. IRFs are key components of the activation of innate antimicrobial response pathways and also contribute to crossroad decisions within T cell differentiation. IRF3 restrains Tc17 differentiation by directly interacting with RORγt through its IRF interaction domain and thereby preventing the binding of RORγt to the *Il17* promoter [148]. In contrast, IRF4 promotes the generation of Tc17 and Th17 cells. *Irf4* deletion prevents the generation of Tc17 and Th17 cells due to lower expression of the Tc17-promoting transcription factors *Rorc* and *Rora*, as well as lower expression of *Il23r* and *Il21*, but higher expression of the Tc17-repressing transcription factor *Eomes* [137]. In sum, the development and maintenance of Tc17 cells not only depend on the presence of specific cytokines but also require specific transcriptional adaptations.

### Tc17 plasticity

Tc17 cells are a highly plastic cell type, especially towards Tc1 or Tc2 fates. CTLA-4 deficiency and reactive oxygen species/IL-2 signaling have both been found to skew Tc17 cell differentiation towards a Tc1 fate with increased IFN-γ, TNF-α, perforin, and granzyme production as well as increased EOMES, T-BET, and BLIMP-1 expression [136, 139, 146, 158]. In contrast, in the context of tissue injury, murine Tc17 cells can acquire a Tc2 phenotype with secretion of IL-5 and IL-13 and expression of GATA3 upon exposure to IL-1, IL-18, and IL-33 [159]. Despite this plasticity, stable Tc17 phenotypes have been described in the setting of murine fungal infection, where Tc17 cells form a TCF1^hi^, TBET^low^, and EOMES^low^ IL-17-producing memory cell population that persists over long periods of time [41, 160–162]. In summary, Tc17 cells are known to be highly plastic and can differentiate into Tc1 or Tc2 cells in specific cytokine milieus, but can also maintain a stable Tc17 phenotype. Since the transfer of Tc17 cells is currently being evaluated in adoptive cell therapy settings to improve anti-cancer immunity [139, 163, 164], this high degree of plasticity needs to be considered.

## Tc17 cells in infectious diseases

### Fungal infection

One hallmark task of type 17 immunity is defense against fungal infections. This is corroborated by the clinical observation that immunodeficient patients with defects in IL-17A and IL-17F signaling due to genetic deficiency of the IL-17 receptor subunit IL-17RA, deficiency in IL-17F or mutations in the downstream adaptor protein NFκB activator 1 (ACT1) often suffer from chronic mucocutaneous candidiasis (CMC) [165–167]. Moreover, patients with dominant-negative mutations in STAT3 show diminished frequencies of IL-17A- and IL-22-producing T cells, and also frequently develop CMC [168]. Furthermore, mucocutaneous candidiasis is a known complication of anti-IL-17A antibody therapy [169], highlighting the importance of IL-17 for defense against fungal infections.

In line with this, IL-17RA KO mice are highly susceptible to local and systemic fungal infection [11, 170]. Mechanistically, IL-17 receptor signaling is responsible for the timely and localized migration of neutrophils to infected organs and for the production of antimicrobial peptides and chemokines [11, 170, 171]. As an additional mechanism for the control of fungal infections, IL-17A has been shown to be necessary for the development of functional NK cells that are crucial for antifungal defense [172].

Historically, Th17 cells were identified as the key producers of IL-17A in anti-fungal protective immunity [11, 166, 173]. Notably however, in a mouse model of local fungal infection using CD4^+^ T cell-deficient mice, IL-17A-secreting CD8^+^ T cells were able to compensate for the lack of Th17 cells and provided protection against oropharyngeal candidiasis [174]. Furthermore, fungal vaccination in CD4^+^ T cell-deficient mice led to the development of vaccine immunity mediated by Tc17 cells. These vaccine-elicited Tc17 cells were able to protect the CD4^+^-deficient hosts from lethal fungal pneumonia, persisted and provided long-lasting memory [41, 160]. Thus, Tc17 cells are sufficient to compensate for the lack of Th17 cells in the setting of fungal infection.

### Viral infection

Compared to fungal infections, the role of Tc17 cells in viral infections is less understood. In mouse models of acute viral infection (influenza virus, vaccinia virus), virus-specific Tc17 cells expand, can acquire a cytotoxic phenotype, and provide protection from lethal infection [131, 136]. In influenza infection, this was associated with enhanced migration of neutrophils into the infected lung [131]. However, mice lacking IL-17A due to genetic deficiency or antibody-mediated depletion had increased viral burden but were still able to clear the virus [136, 175, 176], indicating that IL-17A is enhancing anti-viral immunity but is not necessary for viral clearance. Additionally, *in vitro* polarized Tc17 cells can acquire an IFN-γ-secreting Tc1 phenotype after adoptive transfer and clear vaccinia virus infection [136].

*Tbx21* and *Eomes* double KO CD8^+^ T cells acquire a type 17 transcriptional profile during acute LCMV infection with the Armstrong strain. This is associated with CD8^+^ T cell-dependent inflammation and wasting syndrome accompanied by multi-organ infiltration of neutrophils in the double KO mice [151]. These findings highlight that Tc17 cells can drive immunopathology in viral infections, especially manifested in tissues, in accordance with their importance in immune-mediated tissue diseases such as IBD and psoriasis.

In humans with chronic HCV infection, the frequency of polyfunctional Tc17 cells able to coproduce IL-10, IFN-γ, or IL-21 alongside with IL-17A in the peripheral blood was found to be negatively correlated with the degree of hepatic damage, while the frequency of mono-functional Tc17 cells positively correlated with hepatic damage [177]. In contrast, in the liver of patients with chronic HCV infection and advanced fibrosis, Tc17 cells as well as IFN-γ-coproducing Tc17 cells were found to be increased compared to the peripheral blood of these patients [27, 178]. The role of Tc17 cells for the progression of liver fibrosis is still unclear: IL-17A can contribute to the progression of liver fibrosis by activating hepatic stellate cells [179]. However, the stage of fibrosis in patients with chronic HCV infection was inversely correlated with the frequency of intrahepatic IL-17A/IFN-γ-producing CD8^+^ T cells [27]. These seemingly contradictory findings might be explained by the presence of other IL-17A-producing cell types [179–182]. The literature regarding Tc17 cells in HIV-infected individuals is still inconclusive. While HIV-specific Tc17 cells have been described, observations on overall frequencies of Tc17 cells in HIV-infected individuals compared to control donors are so far variable [183, 184].

In summary, virus-specific Tc17 cells have been identified in both murine and human viral infections, and there is evidence that Tc17 cells enhance the antiviral immune response in murine infections. Importantly, Tc17 responses in viral infections are associated with increased tissue immunopathology.

### Bacterial infection

Consistent with the pathognomonic role of IL-17 in barrier tissue immunity, elevated levels of IL-17A have been described in the gastric epithelium of humans infected with *Helicobacter pylori* and were associated with secretion of IL-8 by gastric epithelial cells, which in turn promoted neutrophil chemotaxis [185]. Furthermore, in mouse models, an important role of IL-17A and IL-17 receptor signaling in the defense against different bacteria like *Streptococcus pneumoniae*, *Bordetella pertussis*, and *Klebsiella pneumoniae* has been identified [10, 186–188]. IL-17A enhances the migration of neutrophils to the site of infection as well as their phagocytic activity [186] and promotes the eradication of intracellular bacteria like *Chlamydia muridarum* by increasing the activity of the inducible nitric oxide synthase [189]. Most of these IL-17 effects have been attributed to the activity of Th17 cells [186–188], have been demonstrated in mouse models, and suggest that the field is only starting to understand the role of Tc17 cells in the defense against bacterial infections in barrier tissues.

## Tc17 cells in cancer

Tc17 cells have been comparatively well characterized in gastroenterological malignancies like gastric cancer, bile duct cancer, and hepatocellular carcinoma (HCC). While there is conflicting data on the frequency of circulating Tc17 cells in patients with gastric cancer [190–192], it has repeatedly been shown that Tc17 cells accumulate in the tumors [190, 192, 193]. Increased intra-tumoral Tc17 cell frequencies are associated with tumor progression and predict a lower overall survival and disease-free survival [190–192]. Tc17 accumulation has been described to occur upon production of IL-6, IL-1β, and IL-23 by tumor-activated monocytes [192]. Intratumoral Tc17 cells themselves then induce the production of CXCL12 by the tumor cells, which causes the recruitment of myeloid-derived suppressor cells and consecutive suppression of cytotoxic CD8^+^ T cell responses [192]. Single-cell RNA sequencing additionally revealed increases in receptor expression for Tc17-derived cytokines like *IL17RA/IL17RC* and *IL22RA1* in gastric cancer cells and it has been hypothesized that these cytokine-receptor interactions promote tumor growth [193].

In accordance with findings in gastric cancer, Tc17 cells have been found to be enriched within HCC, and localize mainly to the tumor margin compared to both intra-tumoral tissue and surrounding non-tumor liver tissue [194, 195]. Most of these Tc17 cells in the tumor margin are able to produce IFN-γ in addition to IL-17A, as opposed to the dominant subset of circulating Tc17 cells in HCC patients, which do not coproduce IFN-γ [194]. Furthermore, intra-tumoral Tc17 cells also express CCL20, CCR4, CCR6, and CXCR6 which have previously been associated with tumor progression in HCC patients [196–198]. Suggested mechanisms for a contribution of Tc17 cells to an immunosuppressive tumor microenvironment include the recruitment of regulatory T cells (*T*_*REG*_) via the CCR6-CCL20 axis indicated by increased levels of CCL20 in HCC samples, especially in those infiltrated with IFN-γ-negative Tc17 cells [195]. Overall, the high intra-tumoral density of IL-17A-producing cells and increased intra-tumoral frequency of IFN-γ-negative Tc17 cells were associated with poorer prognosis in HCC [195, 199]. In patients with bile duct cancer, an elevated frequency of RORγt and T-bet co-expressing CD8^+^ T cells in the peripheral blood was described and Tc17 cells were characterized by a terminal differentiation phenotype with increased TCR signaling despite increased expression of the inhibitory receptors LAG3 and TIM3 [200].

In sum, the current literature points towards an association of Tc17 cells with tumor progression in humans, contrary to data generated in mouse models: Transfer of *in vitro* polarized tumor-reactive Tc17 cells into tumor-bearing mice led to enhanced anti-tumor effects compared to control unpolarized CD8^+^ T cells. Notably, these *in vitro* polarized transferred Tc17 cells differentiated into IFN-γ-producing effector cells [158] similar to observations in murine viral infection. Therefore *in vitro* differentiated Tc17 cells might not recapitulate all features of *in vivo* differentiated, disease-associated Tc17 cells and murine and human Tc17 cells appear to differ in their anti-tumor function.

## Tc17 cells in immune-mediated diseases

### Psoriasis

Consistent with the established role of IL-17 in barrier tissue immunity, in healthy human and primate skin, as well as in wild-caught mice, the presence of Tc17 cells is physiological and considered part of the equilibrium forming barrier immunity [201, 202]. In contrast, specific pathogen-free (SPF) mice have reduced Tc17 frequencies in the skin at baseline [201], but colonization of SPF mice with defined skin commensals like *Staphylococcus epidermidis* induces accumulation of CD8^+^ and Tc17 cells in the skin [201, 202].

Psoriasis is a chronic disease of the skin affecting ~1–2% of the population and is considered to be the most frequent immune-mediated disorder in humans [203, 204]. Most studies refer to plaque psoriasis, which is the most common form of the disease [203, 204]. The frequency of Tc17 cells in the peripheral blood of psoriasis patients correlates with the clinical severity of the disease [205]. Within psoriatic lesions, Tc17 cells are increased compared to the skin of healthy donors [206–209] and the IL-23/IL-17 axis has been shown to sustain psoriatic plaques in humans and mice [210–212]. The antigens targeted by Tc17 cells are presented by the main psoriasis risk gene HLA-Cw6 [213] and other HLA-class I alleles and include the antimicrobial peptide LL-37 (expressed by keratinocytes) [214], the melanocyte-derived antigen ADAMTS-like protein 5 [215], and keratin 17 [216].

Interestingly, in human skin, CD49a expression of CD8^+^ T cells differentiates between IFN-γ and IL-17 production, with CD49a^+^ T cells preferentially producing IFN-γ and CD49a^-^ T cells preferentially producing IL-17. CD49a^-^ CD8^+^ T cells isolated from psoriatic lesions mainly generate IL-17 responses and promote local inflammation [63]. Depleting CD8^+^ T cells prevents psoriasis development in mouse models, indicating that CD8^+^ T cells are already necessary at the early stages of the disease [217].

Around 20% of psoriasis patients develop psoriasis arthritis [218], which in contrast to rheumatoid arthritis is associated with the presence of Tc17 cells in synovial fluid [219, 220], suggesting the specific disease pathogenesis rather than localization of the inflammation determines the presence of Tc17 cells.

Based on this evidence, monoclonal antibodies targeting the IL-17 program have made their way into clinical practice: Treatment of psoriasis vulgaris with secukinumab (targeting IL-17A) or ustekinumab (targeting IL-12/IL-23) led to a rapid reduction of psoriasis symptoms in clinical studies, corroborating the detrimental role of the IL-23/IL-17-axis in the pathogenesis of psoriasis [221, 222]. Furthermore, ixekizumab (anti-IL-17A), brodalumab (anti-IL-17RA), and risankizumab (targeting IL-23A) have proven to be effective [13, 223, 224]. More recently, bimekizumab, a bispecific antibody targeting both IL-17A and IL-17F has been approved [225–227]. Interestingly, dual blockade of IL-17A and IL-17F led to a better clinical response compared to blockade of IL-17A alone with secukinumab, but was associated with a higher rate of oropharyngeal candidiasis [227]. Another dual-inhibitor strategy was tested in clinical trials with COVA322, which inhibits both IL-17A and TNF-α [228], but had to be terminated because of its safety profile (NCT02243787). In Asia, an antibody targeting CD6 expressed by Tc17 cells, itolizumab, is in clinical use for the treatment of psoriasis with the rationale of modulation of CD6-dependent signaling pathways in Tc17 cells [4, 43, 50, 229].

### Multiple sclerosis

MS is the most prevalent chronic inflammatory disease of the central nervous system [230]. Early studies described *IL-17A-*expressing immune cells in the cerebrospinal fluid (CSF) of MS patients [231]. IL-17A production was initially mainly attributed to Th17 cells and their pathogenic role was comprehensively characterized in both patients and murine models of MS [232–235]. Other work then found the frequency of Tc17 cells to be increased in the CSF of MS patients compared to their peripheral blood, and compared to the CSF of control patients [137, 236, 237] and demonstrated a positive correlation between the Tc17 frequency in CSF and the level of disability [238], suggesting a causative link between Tc17 cells and the progression of MS. Furthermore, Tc17 cells were found to be infiltrating the CNS tissue and to be present in both acute and chronic demyelinating lesions of MS patients [233]. In relapsing-remitting MS (RRMS), the most common form of MS, higher frequencies of Tc17 cells, also able to produce IFN-γ and TNF-α, have been described in the peripheral blood in the relapse phase of the disease compared to the remission phase [239].

Murine models of IL-17-deficiency such as *Irf4*-deficient mice that fail to generate Tc17 and Th17 cells and IL-17A-deficient mice are resistant to induction of experimental autoimmune encephalitis (EAE), a common murine model for MS [137, 240, 241]. Interestingly, an adoptive transfer of either wild-type (WT) CD4^+^ or WT CD8^+^ T cells alone into *Irf4*-deficient mice failed to evoke EAE symptoms, only the combined transfer of both WT CD4^+^ and WT CD8^+^ T cells induced the disease. Mechanistically, IL-17A production by CD8^+^ T cells was necessary to induce differentiation and CNS infiltration of Th17 cells and subsequent development of EAE, suggesting that CD8^+^ Tc17 cell help promotes Th17 cell pathogenicity [137].

Currently, more than a dozen disease-modifying drugs to slow down the progression of MS and prolong relapse-free periods are available [230]. No effects of IFN-β, glatiramer acetate, or vitamin D supplementation on the frequency of Tc17 cells in the peripheral blood of MS patients could be detected [237]. In contrast, responders to treatment with dimethyl fumarate (DMF) had a significantly increased frequency of peripheral blood Tc17 cells before treatment initiation compared to non-responders. Clinical and radiological response in this subgroup of MS patients was in turn then linked to a marked decrease in Tc17 cells. Notably, the frequencies of CD4^+^ Th17 cells remained unaffected in responders and non-responders [146]. This points towards a prognostic role of Tc17 cells in the therapeutic response of MS patients and could support personalized therapy approaches. Mechanistically, in the EAE mouse model, DMF has been demonstrated to reduce Tc17-dependent Th17 pathogenicity by reducing IL-17A production by Tc17 cells [146].

Murine studies suggested that the administration of anti-IL-17A antibodies was especially efficient to prevent relapse in a relapsing-remitting model of EAE, while their administration at the peak of the disease had only limited effect [242]. In patients with RRMS, treatment with the IL-17A antibody secukinumab also reduced MR-detectable lesion activity in a proof-of-concept study without notable serious adverse events [243]. A subsequent placebo-controlled, randomized, phase II trial of secukinumab (NCT01874340) was however terminated based on the availability of another human anti-IL-17 antibody, CJM112, which is currently in a phase II trial [13, 244, 245]. In sum, these data suggest that targeting the IL-17 pathway might be a promising therapeutic direction in MS.

### Inflammatory bowel disease

The intestinal immune system is tasked with the challenge of maintaining barrier function and defense against infection while simultaneously tolerating a vast number and variety of intestinal microbiota [246]. In IBD, a disease group comprising Crohn’s disease (CD) and ulcerative colitis (UC), a complex interplay of genetic and environmental factors and impaired intestinal barrier function predisposes individuals to the development of an excessive immune response with acute and chronic inflammatory processes. This leads to further epithelial damage, impaired barrier function, and complications such as abscess formation, stenosis, and increased risk of malignancy [114, 247, 248]. In both CD and UC, IL-17A levels are increased in the serum and mucosa of patients, compared to ischemic or infectious colitis or healthy controls [249]. The involvement of Th17 cells has been previously described [250, 251]. More recent studies have focused on the role of Tc17 cells.

In both healthy controls and IBD patients, Tc17 frequencies are higher in the intestine than in the peripheral blood, consistent with a role in the maintenance of mucosal immune homeostasis [111]. Increased inflammation was found to be associated with Tc17 cell expansion in UC [252]. In accordance with this, our group has published an enrichment of IL-17-producing CD8^+^ T cells with a potentially targetable signature in the peripheral blood and intestinal tissue of patients with active CD [4]. We found this enrichment to be mainly due to conventional αβ-CD8^+^ Tc17 cells rather than unconventional T cell subsets. Disease-associated Tc17 cells produced mainly IL-17A and only small amounts of IL-17F, and expressed a distinct immune signature of CD6^+^, CD39^+^, CD69^+^, PD-1^+^, CD26^+^, CD161^+^, and CD27^low^ which was also linked to flare-free survival of CD patients [4]. Interestingly, in contrast to the increase in IL-17^+^ CD8^+^ T cells, neither IFN-γ^+^ CD8^+^ T cells nor TNF-α^+^ CD8^+^ T cells were significantly increased in the peripheral blood of CD patients. While the total population of IL-17^+^ CD8^+^ T cells was increased in active disease, their polyfunctionality profile did not change in active disease with a preferential coproduction of TNF-α over IFN-γ possibly intrinsically linked to the Tc17 differentiation program. Targeting CD6 reduced the production of proinflammatory cytokines by peripheral blood and intestinal Tc17 cells *in vitro* [4]. In accordance with human data, IL-17^+^ CD8^+^ T cells have been linked to the development of severe colitis in mice [253]. A further mechanistic link between Tc17 cells and dysregulated microbiota in IBD patients could be short-chain fatty acids (SCFAs): Some SCFAs were able to increase expression of IFN-γ and granzyme B, thereby increasing the cytotoxic activity of murine Tc17 cells *in vitro* [254].

In analogy to the successful targeting of the IL-17 axis in psoriasis, the anti-IL-17A antibody secukinumab and the IL-17R targeting antibody brodalumab were evaluated in clinical trials with IBD patients, but these trials unexpectedly had to be terminated. While secukinumab treatment was inefficient and led to an increased rate of infections, treatment with brodalumab was not efficient and even caused disease exacerbation in a disproportionate number of CD patients [255, 256]. Aside from the previously discussed role of IL-17 in the defense against fungal infections, this might be explained by the importance of IL-17 for the maintenance of gastrointestinal barrier integrity: IL-17A-dependent signaling regulates the tight junction protein occludin, and subsequently, IL-17 neutralization increases gut permeability in a DSS (Dextran sulfate sodium)-induced colitis mouse model [257, 258]. Furthermore, for Th17 cells, an autoregulatory loop limiting pathogenicity through IL-17 effects has been described and might have been therapeutically impaired [259].

In contrast to selective targeting of the cytokine IL-17, combined targeting of the IL-23/IL-17 axis with ustekinumab, a monoclonal antibody targeting the shared p40 subunit of IL-12 and IL-23, is an effective treatment strategy in IBD [260]. While ustekinumab is considered to inhibit the differentiation of Th1 and Th17 cells, it has also been shown to affect the differentiation of T follicular helper cells [261]. Another novel drug targeting the IL-23/IL-17 axis via targeting of an IL-23 subunit is risankizumab, which binds to the p19 subunit of IL-23, and has been shown to be effective in CD [262, 263]. In addition to affecting the Tc17 program, IL-23 triggers other pro-inflammatory processes such as reduced T_REG_ function and increased T cell production of TNF-α and GM-CSF [258, 264]. Interestingly, protective IL-17A secretion by γδ T cells was not impaired in settings of IL-23 inhibition [257].

In summary, these clinical studies suggest that targeting the IL-17 axis, but not necessarily selectively targeting the cytokine IL-17 itself can be a viable strategy in the treatment of IBD. Therefore, further expanding our knowledge of how Tc17 cells are generated and how their function is regulated *in vivo* might allow us to identify novel therapeutic targets.

## Conclusion

The last two decades have identified IL-17-producing T cells as uniquely polarized immune cells at the center of many immune-mediated diseases such as IBD. Targeting the IL-17 pathway is an effective treatment strategy for a variety of diseases. While most studies have focused on CD4^+^ Th17 cells as the classical IL-17-producing T cell population, a growing body of evidence suggests that CD8^+^ Tc17 cells are similarly involved in the pathogenesis of many immune-mediated diseases and infections. Due to the diversity of conventional and unconventional CD8-expressing cell types that can produce IL-17, such as αβ Tc17 cells, γδ T cells, MAIT cells, and NKT cells, the pathogenic versus protective role of IL-17-producing CD8^+^ T cells is still incompletely understood. Particularly in humans, the environmental and transcriptional requirements for the generation of Tc17 cells are not yet well defined. Future mechanistic studies are needed to allow a deeper understanding of this novel T cell population and will help to expand on currently available targeted therapies.

## Data Availability

No new data were generated in this study.

## Abbreviations:

ADAMTS: a disintegrin and metalloproteinase with thrombospondin motifs
AhR: aryl hydrocarbon receptor
ALCAM: activated leukocyte cell adhesion molecule/CD166
ATP: adenosine triphosphate
BCL-x_L_: B-cell lymphoma-extra large
Blimp-1: B lymphocyte-induced maturation protein-1
CCL: C-C motif chemokine ligand
CCR: C-C motif chemokine receptor
CD: Crohn’s disease
CMC: chronic mucocutaneous candidiasis
CNS: central nervous system
CSF: cerebrospinal fluid
CTLA-4: cytotoxic T-lymphocyte-associated protein 4
CXCL: chemokine (C-X-C motif) ligand
CXCR: chemokine (C-X-C motif) receptor
DMF: dimethyl fumarate
DSS: dextran sulfate sodium
EAE: experimental autoimmune encephalomyelitis
ENTPD1: ectonucleoside triphosphate diphosphorylase 1/CD39
Eomes: eomesodermin
FICZ: 6-formylindolo[3,2-b]carbazole
G-CSF: granulocyte colony-stimulating factor
GM-CSF: granulocyte-macrophage colony-stimulating factor
HCC: hepatocellular carcinoma
HCV: hepatitis C Virus
HIV: human immunodeficiency virus
HLA: human leukocyte antigen
IBD: inflammatory bowel disease
IFN-γ: interferon-γ
Ikzf: Ikaros family zinc finger protein
IL: interleukin
IRF: interferon regulatory factor
JAK: Janus kinase
KO: knockout
LAG3: lymphocyte-activation gene 3
LAT1: L-type amino acid transporter 1
LLT1: lectin-like transcript 1
MAIT cell: mucosa associated invariant T cell
MHC: major histocompatibility complex
mRNA: messenger RNA
MS: multiple sclerosis
NFκB: nuclear factor κB
NKT cell: natural killer T cell
PD-1: programmed cell death protein 1
PLZF: promyelocytic leukemia zinc finger
RORγt: retinoic acid receptor-related orphan receptor-γt
RRMS: relapsing remitting MS
S1PR1: sphingosine-1-phosphate receptor 1
SCFAs: short-chain fatty acids
SPF: specific pathogen free
STAT: signal transducer and activator of transcription
T-bet: T-box expressed in T cells
Tc1: cytotoxic T lymphocyte
Tc17: interleukin 17-producing CD8^+^ T cell
Tc2: Type 2 CD8^+^ T cell
Tc9: interleukin-9-producing CD8^+^ T cell
TCF-1: T cell factor-1
TCR: T cell receptor
TGF-β: transforming growth factor-β
Th1: T helper 1 cell
Th17: T helper 17 cell
Th2: T helper 2 cell
Tim3: T cell immunoglobulin and mucin-domain containing 3
TNF-α: tumor necrosis factor-α
Treg: regulatory T cell
TRM: tissue resident memory cells
UC: ulcerative colitis
WT: wild type
